# Αcute visceral cysticercosis caused by *Taenia hydatigena* in lambs: ultrasonographic findings

**DOI:** 10.1186/s13071-020-04439-x

**Published:** 2020-11-11

**Authors:** Andrea Corda, Giorgia Dessì, Antonio Varcasia, Silvia Carta, Claudia Tamponi, Giampietro Sedda, Mauro Scala, Barbara Marchi, Francesco Salis, Antonio Scala, Maria Luisa Pinna Parpaglia

**Affiliations:** 1 Dipartimento di Medicina Veterinaria, Università degli Studi di Sassari, Sassari, Italy; 2 Facoltà di Medicina e Chirurgia, Università degli Studi di Sassari, Sassari, Italy; 3 Area Socio-Sanitaria Locale, Oristano, Italy; 4 Veterinary practitioner, Sassari, Italy

**Keywords:** Metacestodosis, *Taenia hidatigena*, Acute form, Sheep, Diagnosis

## Abstract

**Background:**

Cysticercosis caused by *cysticercus tenuicollis* is a metacestode infection that affects several species of ungulates. It is caused by the larval stage of *Taenia hydatigena*, an intestinal tapeworm in dogs and wild canids. In the intermediate host, the mature cysticerci are usually found in the omentum, mesentery, and peritoneum, and less frequently in the pleura and pericardium. The migrating larvae can be found mostly in the liver parenchyma causing traumatic hepatitis in young animals. Most infections are chronic and asymptomatic, and are diagnosed at the abattoir. The acute form of infection is unusual in sheep and reports of death in lambs are rare.

**Methods:**

In March 2018, fifteen female lambs presented anorexia, weakness, lethargy, and death secondary to acute visceral cysticercosis. Twelve of them underwent hepatic ultrasonography. Examinations were performed on standing or left lateral recumbent animals.

**Results:**

Livers of affected animals presented rounded margins and a thickened, irregular and hyperechoic surface. Hepatic parenchyma appeared to be wholly or partially affected by lesions characterized by heterogeneous areas crossed by numerous, irregular, anechoic tracts ranging from 1 to 2 cm in length and 0.1 to 0.2 cm in width. Superficial and intraparenchymal cystic structures were also visualized. The presence of lesions was confirmed by anatomopathological examination, and *T. hydatigena* cysticerci was identified by morphological and molecular characterization of isolates.

**Conclusions:**

Our results highlighted that hepatic ultrasonography is effective for an *intra-vitam* diagnosis of acute cysticercosis in lambs.
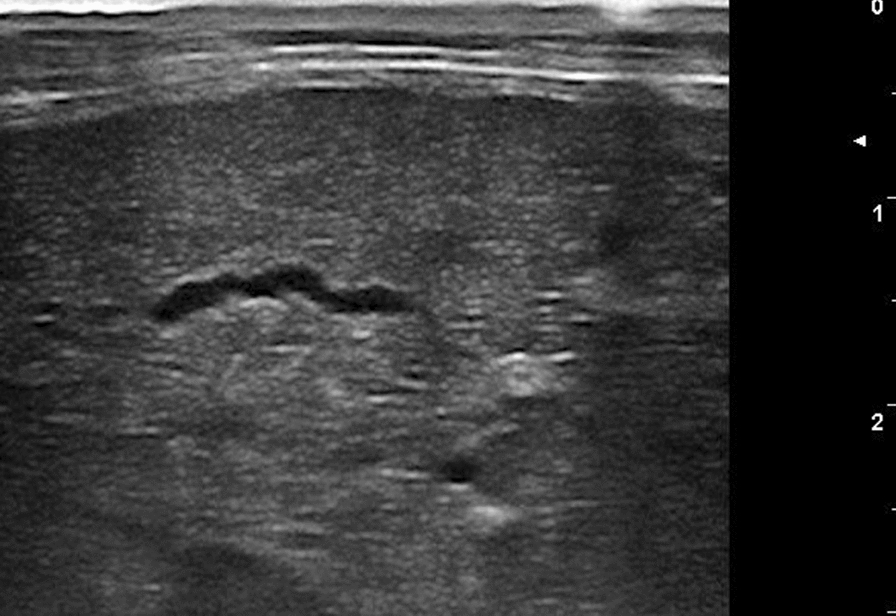

## Background

The metacestode *cysticercus tenuicollis* is the larval stage of *Taenia hydatigena,* a cosmopolitan tapeworm in dogs and wild canids [1–3]. The development of *T. hydatigena* requires two distinct hosts to complete its life-cycle. The adult parasites reside in the intestine of the definitive hosts such as dogs and other carnivores such as foxes, wolves, jackals, lynx, raccoons, bears and cats [4]. The intermediate hosts, generally small ruminants and, less frequently pigs, cattle, deer and other wild species, get infected by ingesting eggs from contaminated pasture [5–7]. After the eggs hatch in the small intestine of the intermediate hosts, developing cysticerci migrate to reach the liver, and occasionally other organs such as the lungs and kidneys [8].

Migrating larvae can be found in the liver parenchyma of lambs and piglets within 7–10 days post-infection [9, 10]. Mature cysticerci are usually found in the omentum, mesentery, peritoneum and, less frequently, in the pleura and pericardium [8–13]. Clinical signs in intermediate hosts vary according to the severity of infection. Most infections are chronic and asymptomatic, and are not usually identified until slaughter [14].

In severe infections, the migration of a large number of larvae causes severe traumatic hepatitis, peritonitis and even pneumonia, leading to clinical signs and even death [8, 12, 15]. A mortality rate of 19.0% has been described in lambs due to massive hepatic and pulmonary infections [12].

Cysticercosis caused by *cysticercus tenuicollis* is widespread in sheep in Italy, where an overall prevalence of 14.6% has been reported in lambs. In Sardinia, the economic loss related to *cysticercus tenuicollis* infection has been estimated at €330,000 per year [16]. Although several attempts have been made for the ante-mortem diagnosis of *T. hydatigena* cysticercosis by serological tests [17–20], a final diagnosis of the disease is currently only possible by analyzing the cysts after the animal has been slaughtered.

Although ultrasonography is effective in diagnosing parasitic infections in animals, including other metacestodoses such as cystic echinococcosis and coenurosis [21–30], to the best of our knowledge, there are no reports describing the ultrasonographic findings of *cysticercus tenuicollis* infection in small ruminants.

In this paper we describe the clinical manifestation, ultrasonographic findings, anatomopathological results and molecular characterization of lambs affected by acute visceral cysticercosis by *T. hydatigena.*

## Methods

In March 2018, fifteen female Sarda lambs (native to Sardinia, Italy), from a flock of 100 individuals, four months old, weighing from 15 to 20 kg, presented various degrees of anorexia, lethargy, weakness and death.

The animals had not previously received parasitological treatments. Necropsy on three dead animals revealed acute visceral cysticercosis as the cause of death. The twelve surviving animals underwent physical and coprological examination as well as hepatic ultrasonography.

Coprological examination was carried out using the FLOTAC^®^ technique: specific gravity (s.g.) 1350, and eggs per gram (EPG) or oocysts per gram (OPG) were obtained using a zinc sulphate solution [31, 32]. The ultrasonographic examinations were performed by an experienced operator (AC) with a portable ultrasound unit (My Lab Alpha, Esaote, Florence, Italy) equipped with two multifrequency linear (SL1543; 3-13 MHz) and microconvex (SC3123; 4-9 MHz) probes. Images and video obtained with both probes were acquired and stored for offline review.

Examinations were performed on standing or left lateral recumbent unsedated lambs. The liver was visualized by placing the probe in the cranial right hypochondrium, caudally to the last rib or between the right intercostal spaces [21]. The hepatic parenchyma was examined by transverse and longitudinal sections. Based on ultrasonographic findings, the severity of infection was ranked into 3 classes: severe, when the lesions affected the whole hepatic parenchyma; moderate, when the lesions occupied most of the parenchyma; and mild, when the extent of the injuries was less than the normal parenchyma. The appearance of the liver surface (regularity and thickness) and edges (sharp or rounded) were also examined. During the ultrasonographic examination, B-lines on the diaphragmatic surface of the right lung were also observed along with free abdominal fluid. The animals that died within 24 hours of the ultrasound scan underwent necropsy. Liver and lung parenchyma samples were fixed with 10% neutral buffered formalin solution and embedded in paraffin wax. Sections were stained with hematoxylin and eosin for histopathological examination.

Morphological identification of the parasites was performed according to the previously reported keys [33]. Molecular identification was then performed in order to confirm morphological diagnosis according to Scala et al. [16].

## Results

On physical examination, the lambs presented pale mucous membranes (*n* = 7), body condition score < 3 (*n* = 5), depression and unwillingness to move (*n* = 5), inspiratory crackles on pulmonary auscultation (*n* = 4), hypothermia (n=3), sub-icteric mucous membranes (*n* = 3), respiratory distress (*n* = 3), hyperthermia (*n* = 2), sternal recumbency (*n* = 2), and abdominal distension (*n* = 2).

At coprological examination, eggs of gastro-intestinal strongyles (GIS) were detected (average 60 EPG) in eight of the twelve lambs, an average of 30 EPG of *Nematodirus* spp. were found in four lambs, and all the lambs showed *Eimeria* spp. oocysts (average 180 OPG).

All the examined lambs showed ultrasonographic evidence of liver lesions. On the basis of the ultrasonographic classification, the infection was scored as severe in six lambs, moderate in five, and mild in only one animal. The severity of clinical symptoms was directly related to the extent and severity of liver injuries.

**Fig. 1:**
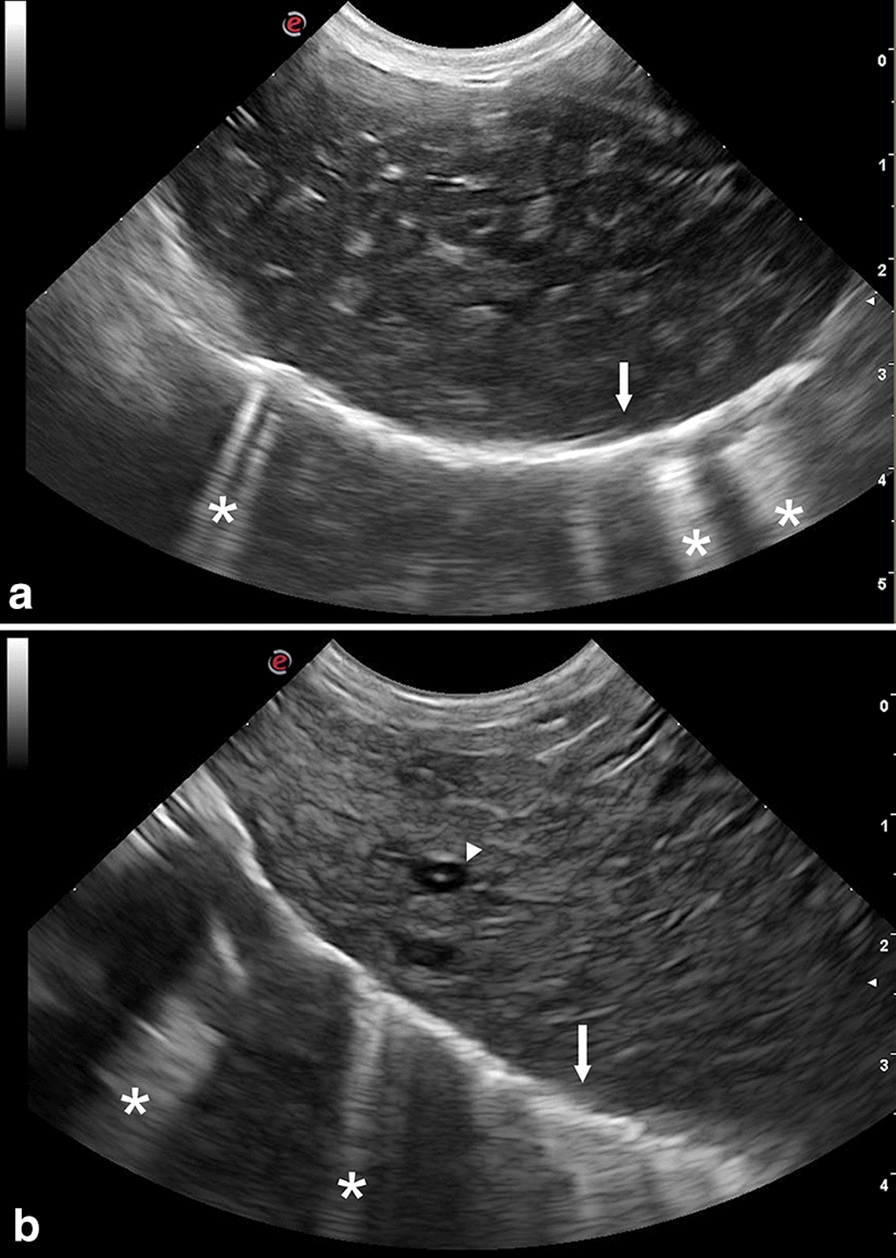
Ultrasonographic appearance of the lamb’s liver affected by acute cysticercosis. **a** Ultrasonographic transversal section of the liver showing a diffusely heterogeneous parenchyma crossed by hypoechoic irregular tracts. The diaphragmatic surface of the liver appears irregular, thickened and hyperechoic (arrow). The lung surface presents several B-lines (asterisks). **b** Ultrasonographic longitudinal section of the liver showing a diffusely heterogeneous parenchyma in which intraparenchymal cysts are visible (arrowhead). The diaphragmatic surface of the liver appears irregular, thickened and hyperechoic (arrow). The lung surface presents several B-lines (asterisks)

**Fig. 2:**
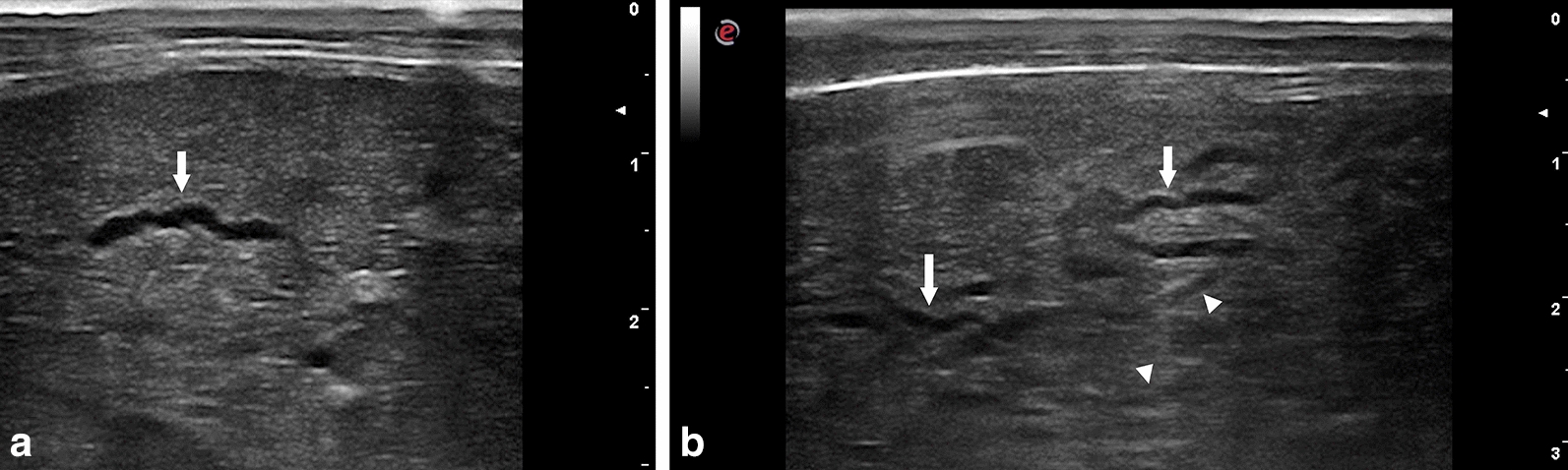
Ultrasonographic appearance of hepatic migratory tracts produced by *cysticercus tenuicollis*. **a** Ultrasonographic aspect of a hepatic intraparenchymal anechoic tract (arrow). **b** Ultrasonographic aspect of several hepatic intraparenchymal anechoic (arrow) and hypoechoic (head arrow) tracts

**Fig. 3:**
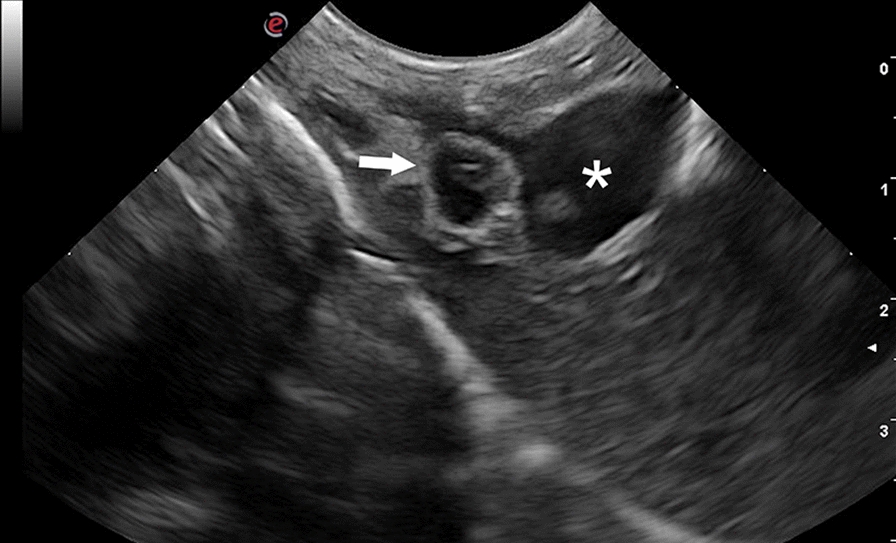
Ultrasonographic longitudinal section of the liver showing a cystic structure containing a hyperechoic mural branching component (arrow) localized along the hepatic visceral surface at the level of the portal vein (asterisk)

**Fig. 4:**
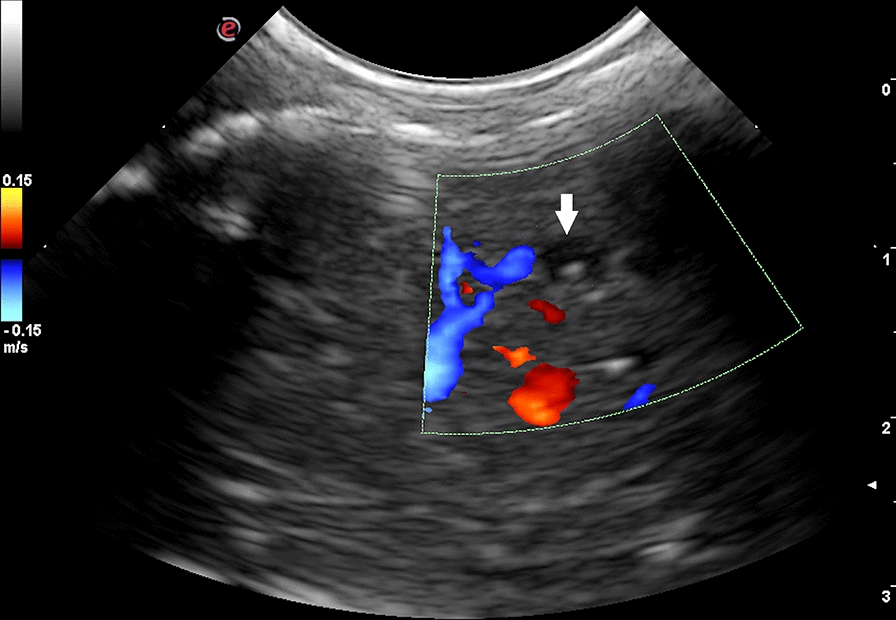
Color Doppler examination of the liver showing an intraparenchymal cyst (arrow)

Hepatic lesions were characterized by heterogeneous echotexture and mixed echogenicity (Fig. 1). The injured areas were crossed by several irregular hypo- or anechoic tracts ranging from 1 to 2 cm in length and 0.1 to 0.3 cm in width (Fig. 2). Five severely infected animals presented cystic structures (from 0.5 to 0.7 cm in diameter) localized along the hepatic surface and characterized by a thick and hyperechoic wall, containing a hyperechoic mural branching component surrounded by anechoic fluid (Fig. 3). They also presented intraparenchymal cysts (from 0.3 to 0.4 cm in diameter) containing a point-like hyperechoic structure surrounded by anechoic fluid (Fig. 1b). A Doppler color examination confirmed the cystic nature of these structures (Fig. 4).

All the lambs with severe infection and three with moderate infection presented rounded liver margins. All the lambs with severe infection and two with moderate infection also presented an irregular, thickened and hyperechoic liver surface. Four lambs with severe and three with moderate liver damage showed enlarged portal lymph nodes. Three animals with severe and one with mild infection presented B-lines on the diaphragmatic surface of the right lung (Fig. 1). Two severely infected lambs also presented evidence of free peritoneal fluid accumulation, which was hypoechoic with suspended echogenic particles. No lamb showed ultrasonographic evidence of biliary system alterations.

Four of the severely infected animals died (*n* = 2) or were euthanized (*n* = 2) for welfare reasons, within 24 hours of the ultrasound and underwent necropsy. The surviving lambs (*n* = 8) underwent medical treatment with oral praziquantel [12]. Two of them, both classified as severely infected, died several days later; however, they were not taken into consideration for this study because too much time had passed since the ultrasound. The other six lambs, five classified as moderately and one as mildly infected, survived the infection and were still alive after one year.

**Fig. 5:**
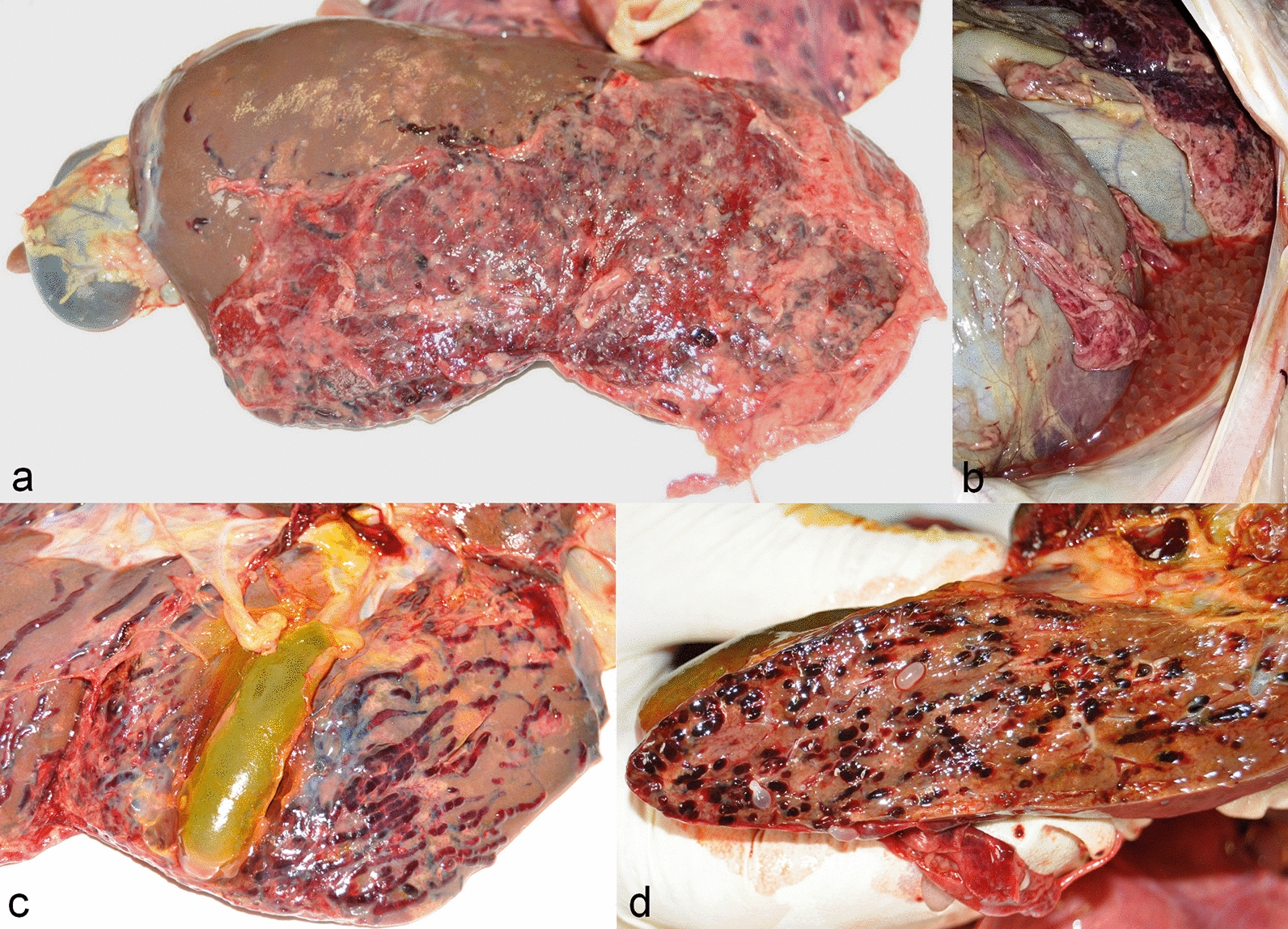
Gross pathology images. **a** Severe perihepatitis with whitish-yellow fibrin depositions on the diaphragmatic liver surface. **b** Free abdominal fluid containing several parasitic cysts. **c** Hepatic surface crossed by multiple hemorrhagic wavy tracts. **d** Hepatic section showing multiple intraparenchymal hemorrhagic tracts and parasitic cysts

The four lambs that underwent necropsy showed similar gross and histopathological changes. Gross pathology revealed hepatomegaly and perihepatitis with whitish-yellow fibrin depositions on the liver surface (Fig. 5a). All the animals presented free fluid (inflammatory exudate) in the abdomen in which parasitic cysts (3–5 mm) were floating (Fig. 5b). The hepatic surface as well as the parenchyma sections were crossed by multiple, 2–3 mm wide, hemorrhagic wavy tracts (Fig. 5c, d). Several parasitic cysts were detected on the surface and inside the parenchyma of the liver and lungs as well as in the mesentery.

Upon histological examination, the larval tracks appeared to be filled with erythrocytes, neutrophils, eosinophils necrotic cells debris and fibrin. Migrating cysticerci were surrounded by erythrocytes, neutrophils, eosinophils, necrotic cells, lymphocytes and macrophages. Similar lesions were observed in the pulmonary parenchyma.

The morphological characteristics of isolated cysts were consistent with those reported in the literature for *T. hydatigena* cysticerci, in different stages of maturation [33]. The morphological identification was confirmed by molecular biology and nucleotide sequences showed a 99% identity with *T. hydatigena* (GenBank: AB792722 and JN831270).

## Discussion

The present report demonstrates that ultrasonography is a useful diagnostic tool to identify and define the extent and severity of hepatic lesions caused by *cysticercus tenuicollis* migration in lambs.

Ultrasonography can be used to directly visualize the parasites and/or to show the lesions caused by them. In this report we have described the ultrasonographic appearance of the hepatic injuries caused by the larval stage migration, as well as the parasite itself. Hepatic lesions were heterogeneous, with mixed echogenicity areas crossed by several hypo- or anechoic irregular tracts. Areas with a non-homogeneous echotexture within the liver parenchyma cannot be considered a specific finding of *cysticercus tenuicollis* infection, as similar alterations have also been described in ovine fascioliasis [34]. However, our results show that this type of hepatic parenchymal injury, which is not associated with alterations in the biliary system, is highly indicative of *cysticercus tenuicollis* infection, especially in endemic regions.

Our results showed that the migratory tracts had variable echogenicity: some were completely anechoic, others were hypoechoic with varying shades of gray. The variability was probably due to the different stage of histological development of the hemorrhagic tracts caused by *cysticercus tenuicollis* migration [11, 13]. The ultrasonographic appearance of intraparenchymal hemorrhagic lesions changes over time: initially they are echogenic and later become hypo- and anechoic [35, 36]. The intra- and extrahepatic alterations visualized by ultrasonography were subsequently confirmed by necropsy: (i) the irregular thickened and hyperechoic liver surface was due to the presence of several superficial tracts and to the perihepatitis; (ii) the rounded liver margin was associated with hepatomegaly [35, 37]; and (iii) the presence of several B-lines on the diaphragmatic surface of the right lung was due to pneumonia secondary to larval migration. In fact, the B-line artifact is a sign of pulmonary interstitial disease and is considered an early sign of pneumonia in ruminants [38, 39].

Hepatic and perihepatic cysts compatible with *cysticercus tenuicollis* were identified in five lambs. They appeared as cystic structures attached to the visceral surface or inside the hepatic parenchyma. Not all the livers of the lambs displayed parasites, probably because in some animals the larvae were not sufficiently mature to be visualized or because gas in the gastrointestinal tract adjacent to the liver made it impossible to visualize the parasites.

Ultrasonographic evidence of intraparenchymal cysts in sheep has also been described in cystic echinococcosis [21, 24, 25]. However, cystic echinococcosis is characterized by the presence of cystic structures ranging from 0.9 to 10.4 cm in diameter, surrounded by normal or moderately heterogeneous parenchyma in which there is no evidence of migratory tracts [21, 24]. In contrast, the intraparenchymal cysts described in this report were always small (maximum 0.4 cm) and surrounded by severely injured tissue.

In our study, due to the animals’ young age, it would have been unlikely to misdiagnose the two parasites. However, differentiating between cystic echinococcosis and cysticercosis in animals older than 18 months can be difficult or impossible when cysts are attached to the liver surface because their appearance under ultrasound may be identical.

Early diagnosis of cysticercosis caused by *T. hydatigena* in live animals is important from an epidemiological point of view because positive animals indicate the level of environmental contamination with other metacestodoses. Although there are differences due to the species, *T. hydatigena* share the same hosts and life-cycle modality with Taeniid species of zoonotic interest such as *E. granulosus* (*sensu lato*) and *T. multiceps* both in domestic and wildlife environments [40–43].

Interestingly, cysticercosis is usually present in the first months of age of the intermediate hosts (as in our case), while diagnosis of coenurosis is usually possible in animals between 4 and 18 months [44], and a diagnosis of cystic echinococcosis is usually obtained in animals older than 18 months, in relation to the growth rates of the cysts, which influence the reliability of the diagnosis. In *E. granulosus* (*sensu lato*), the growth rate is slow and variable and dependent on the species or strains of the parasite [45] and the species of the host and the degree of infection. In sheep, cysts increase by between 1 and 5 cm in diameter per year [46], and from 1–2 mm to 10 mm per year in humans [47]. There is an intraspecific variability in the genetic structure of *T. hydatigena*, which is consistent with biochemical and morphological studies that suggest the existence of other variants of these parasites [48]. It would thus be interesting to understand whether or not there are also different rates of growth of *T. hydatigena* cysticerci.

We believe that cysticercosis by *T. hydatigena*, is not only an economic problem for farmers and the meat industry, but could also be life threatening for young animals, as already reported [16].

Ultrasonography is increasingly used as an *intra-vitam* test to diagnose parasitic diseases in small ruminants, probably because of the widespread use of portable ultrasound equipment by sheep practitioners [29, 49]. Hepatic ultrasonography could be a useful *intra-vitam* test to diagnose *cysticercus tenuicollis* infections in sheep. However, the application of this method could be limited by the need to use high-quality portable ultrasound equipment by experienced staff, which could increase the costs of the examinations. Moreover, it is not clear how sensitive and specific the ultrasonography would need to be in order to reveal mild infections in asymptomatic animals, which represent the majority of cases. Further studies are thus needed to determine whether the method can be used as part of epidemiological surveillance and control programmes.

## Conclusions

The present report demonstrates that ultrasonography can identify and define the extension and the severity of hepatic lesions caused by *cysticercus tenuicollis* in lambs. This technique could be used as an *intra vitam* diagnostic test for *cysticercus tenuicollis* parasitosis in small ruminants.

## Data Availability

All data generated or analyzed during this study are included in this published article.

## Abbreviations

EPG: Eggs per gram
OPG: Oocysts per gram
AC: Andrea Corda
GIS: Gastro-intestinal strongyles
